# Investigating associations between the natural environment and COVID-19

**DOI:** 10.23889/ijpds.v9i5.2772

**Published:** 2024-09-18

**Authors:** Oliver Thwaites, Amy Mizen, Ronan Lyons, Rhiannon Owen, Benedict Wheeler, Richard Fry

**Affiliations:** 1 Swansea University; 2 University of Exeter

## Background

The natural environment offers a plethora of health benefits and may mitigate the risk of contracting SARS-CoV-2. However, they are also sites where interpersonal contact is frequent, amplifying the risk of transmission. Previous research has typically focused on green space or a measure of greenness. Greenness is often associated with fewer COVID-19 infections, whereas associations with green space are mixed. Very few studies have investigated blue spaces. To our knowledge, no studies have investigated associations between greenness, green space, and blue space with COVID-19 for a national population.

## Objectives & Approach

We aimed to investigate the impacts of exposure to the natural environment on the likelihood of testing positive for COVID-19 in small areas across Great Britain. We derived several measures of green space, greenness and blue space using data from Ordnance Survey and the Google Earth Engine. These measures were linked to the COVID-19 Infection Survey in a Trusted Research Environment. We used mixed-effects logistic regression to account for the multiple tests taken by each participant, adding a random effect for participant ID. We fit univariate models for each predictor and multivariate models for each environmental exposure, adjusting for sex, age, ethnicity, household occupancy, rurality, population density and deprivation.

## Results

The survey contained test results from a random and representative sample of over 500,000 participants. Approximately 11 million tests were taken by the cohort across three years.

## Conclusions & Implications

The results of this study will inform planning for the next pandemic.

